# Is 0.01% Atropine an Effective and Safe Treatment for Myopic Children? A Systemic Review and Meta-Analysis

**DOI:** 10.3390/jcm10173766

**Published:** 2021-08-24

**Authors:** Hou-Ren Tsai, Tai-Li Chen, Jen-Hung Wang, Huei-Kai Huang, Cheng-Jen Chiu

**Affiliations:** 1 Department of Medical Education, Medical Administration Office, Hualien Tzu Chi Hospital, Buddhist Tzu Chi Medical Foundation, Hualien 970, Taiwan; melotsai0830@gmail.com (H.-R.T.); terrychen.a@gmail.com (T.-L.C.); 2 School of Medicine, Tzu Chi University, Hualien 970, Taiwan; drhkhuang@gmail.com; 3 Department of Dermatology, Tzu Chi Skin Institute, Hualien Tzu Chi Hospital, Buddhist Tzu Chi Medical Foundation, Hualien 970, Taiwan; 4 Department of Medical Research, Buddhist Tzu Chi General Hospital, Hualien 970, Taiwan; jenhungwang2011@gmail.com; 5 Department of Family Medicine and Medical Research, Hualien Tzu Chi Hospital, Buddhist Tzu Chi Medical Foundation, Hualien 970, Taiwan; 6 Department of Ophthalmology and Visual Science, Tzu Chi University, Hualien 970, Taiwan; 7 Department of Ophthalmology, Hualien Tzu Chi Hospital, Buddhist Tzu Chi Medical Foundation, Hualien 970, Taiwan

**Keywords:** 0.01% atropine, myopia control, axial length, standardized equivalent refraction

## Abstract

Several conflicting results regarding the efficacy of 0.01% atropine in slowing axial elongation remain in doubt. To solve this issue and evaluate the safety of 0.01% atropine, we conducted a systematic review and meta-analysis with the latest evidence. The review included a total of 1178 participants (myopic children). The efficacy outcomes were the mean annual progression in standardized equivalent refraction (SER) and axial length (AL). The safety outcomes included mean annual change in accommodative amplitude, photopic and mesopic pupil diameter. The results demonstrated that 0.01% atropine significantly retarded SER progression compared with the controls (weighted mean difference [WMD], 0.28 diopter (D) per year; 95% confidence interval (CI) = 0.17, 0.38; *p* < 0.01), and axial elongation (WMD, −0.06 mm; 95% CI = −0.09, −0.03; *p* < 0.01) during the 1-year period. Patients receiving 0.01% atropine showed no significant changes in accommodative amplitude (WMD, −0.45 D; 95% CI = −1.80, 0.90; *p* = 0.51) but showed dilated photopic pupil diameter (WMD, 0.35 mm; 95% CI = 0.02, 0.68; *p* = 0.04) and mesopic pupil diameter (WMD, 0.20 mm; 95% CI = 0.08, 0.32; *p* < 0.01). In the subgroup analysis of SER progression, myopic children with lower baseline refraction (>−3 D) and older age (>10-year-old) obtained better responses with 0.01% atropine treatment. Furthermore, the European and multi-ethnicity groups showed greater effect than the Asian groups. In conclusion, 0.01% atropine had favorable efficacy and adequate safety for childhood myopia over a 1-year period.

## 1. Introduction

Myopia is becoming a public health concern with a significant socioeconomic burden affecting 80% to 90% of young adults [1,2,3,4,5,6,7]. By 2050, Holden et al. has predicted that 9.8% of the world’s population would be high myopia cases [8], leading to severe sight-threatening complications, such as glaucoma, myopic macular degeneration, and retinal detachment [9,10,11,12]. Thus, finding an effective and safe treatment to inhibit myopia progression is urgently needed [13].

The efficacy of atropine (a non-selective antagonist of muscarinic acetylcholine receptors) to prevent myopia progression in children has been studied widely. Different concentrations of atropine (0.01% to 1%) have been shown to inhibit myopic progression effectively [14,15,16]. However, high dose atropine has been subject to significant adverse effects such as blurred near vision, photophobia and rebound phenomenon after treatment cessation [16]. Chia et al. [17] have evaluated the change in standardized equivalent refraction (SER) and axial length (AL) after stopping the administration of atropine in a 5-year study and concluded that cessation of 0.1% and 0.5% atropine resulted in a greater degree of myopic rebound, but 0.01% atropine appears to result in less myopic rebound, which led to a more sustained effect of myopia retardation. They also proposed that a daily dose of atropine 0.01% is an effective first-line treatment in children aged 6 to 12 years with documented myopic progression of −0.5 D in the preceding year with few side effects.

Several studies have shown that low dose atropine, especially 0.01%, may slow SER progression with minimal side effects; nevertheless, the effect in inhibiting axial elongation is still inconsistent [18,19,20]. Fu et al. reported that 0.01% atropine significantly reduced myopia progression over a 12-month period as measured by AL when compared with a control group (average 0.14 mm, *p* = 0.004) (19). However, Khanal et al. [21] asserted that 0.01% atropine could not slow the abnormal eye enlargement, thus delaying implementing an effective dose. Li et al. [22] have pointed out that this phenomenon may be due to the sample size among previous studies powered primarily based on SER change and concluded that a larger sample size is needed to detect the difference in AL elongation between the 0.01% atropine and placebo groups. Although one meta-analysis [23] has enrolled seven RCTs to investigate the efficacy of 0.01% atropine in axial elongation, the control group differed among the enrolled studies, which may bias the actual effect of 0.01% atropine. Of note, excessive elongation of the eyeball may increase the risk of subsequent myopia complications [24,25], it is essential to determine whether 0.01% atropine can effectively inhibit axial elongation.

In addition, the most frequently reported side effects of topical atropine include blurred near vision, allergic reaction, and dilated pupil, which may increase the exposure of the lens and retina to ultraviolet light [26]. Although these were short-term and minimal in 0.01% atropine [14,19], it is also worthy of being investigated and compared with other concentrations of atropine in long-term use. Furthermore, the relevant evidence regarding the efficacy of 0.01% atropine compared to placebo continues to accumulate in recent years [19,20,27,28]. Thus, we conducted a rigorous quantitative and systematic summary of the evidence to increase the statistical power and elucidate the conflicting results of 0.01% atropine in childhood myopia. Furthermore, subgroup analysis according to known confounding factors such as different ethnicity, baseline age, and baseline myopia status was conducted to identify the ideal recipients for 0.01% atropine in myopia control.

## 2. Materials and Methods

### 2.1. Study Design

This meta-analysis aimed to survey the efficacy and safety of 0.01% atropine in myopia control. The study was performed per the recommendations made by the preferred reporting items for a systematic review and meta-analysis (PRISMA) statement (Table S1), and the methodology was pre-specified and registered on the INPLASY website (Registration No. INPLASY202140082).

### 2.2. Search Strategy

Studies describing the efficacy of 0.01% atropine in myopia control before June 2021 were identified from the PubMed, Embase, and Cochrane Library databases. No language restrictions were applied. The keywords “0.01% atropine,” “myopia control,” and their synonyms and derivatives were used. Details of the search strategies are described in Table S2. The “related articles” option in PubMed was used to broaden the search results, and all abstracts, studies, and citations retrieved were reviewed. Furthermore, we assessed the reference sections of the retrieved articles to identify other relevant studies. Lastly, relevant studies were retrieved from the ClinicalTrials.gov registry (https://clinicaltrials.gov/, accessed on 27 June 2021) and the International Clinical Trials Registry Platform (ICTRP, https://www.who.int/ictrp/en/, accessed on 27 June 2021).

### 2.3. Inclusion and Exclusion Criteria

Studies were included in the systematic review if: (1) they were randomized control trials (RCTs), cohort studies, or case-control studies; (2) they compared a group treated with 0.01% atropine for myopia control with a control group; (3) the participants with a diagnosis of myopia were younger than 18 years; (4) at least one efficacy or safety outcome relevant to our review was reported in the studies, including the change in SER, AL, accommodative amplitude, and pupil size; and (5) the mean follow-up period was at least one year. We excluded review articles, case reports, case series, and animal or laboratory studies.

### 2.4. Data Extraction

Two authors (H.-R.T. and T.-L.C.) independently extracted the following items: first author, year of publication, study design, number of eyes, baseline SER, baseline AL, follow-up period, drop-out rate, and details of the treatment arm. The efficacy outcomes were the changes in SER and AL per year. The safety outcomes included changes in accommodative amplitude, photopic pupil size, and mesopic pupil size.

### 2.5. Quality Assessment

The methodological quality of the non-randomized studies was assessed using risk of bias in non-randomized studies-of interventions (ROBINS-I) [29], and that of the RCTs was evaluated using the Cochrane Collaboration’s risk of bias assessment tool (RoB v.2.0) [30]. Decisions recorded individually by the reviewers (H.-R.T. and T.-L.C.) were compared, and disagreements were resolved by a third reviewer (C.-J.C.).

### 2.6. Data Synthesis and Statistical Analyses

The effect size of each study was presented as WMD with 95% CIs for continuous outcome measures (SER, AL, accommodative amplitude, mesopic pupil size, and photopic pupil size). When standard deviation data were not applicable, we calculated standard deviations with formulas described in the Cochrane Handbook for Systematic Reviews of Interventions [31]. The pooled estimates and their CIs were calculated using the DerSimonian and Laird random-effects model, considering the heterogeneity of the study populations [31]. The modified HKSJ adjustment was employed to adjust for type I errors and avoid inaccurate CIs as a sensitivity analysis if the included study number of each outcome was less than 10 and the pooled effect was statistically significant [32,33].

The statistical heterogeneity among studies was tested using I^2^ statistics [34]. The statistical heterogeneity was considered significant when the I^2^ statistic was ≥50%. We performed a leave-one-out sensitivity analysis to evaluate each study’s influence on the overall effect by removing studies sequentially. Further, we conducted a subgroup analysis according to the study design, study population, mean age, and mean baseline refraction to explore the potential heterogeneity. The pooled effect sizes were deemed significant when the 95% CI of the mean difference (MD) did not cross zero. All statistical tests were two-sided, and *p*-values <0.05 were considered statistically significant. Outcome data were analyzed using Stata v17 (StataCorp, College Station, TX, USA).

## 3. Results

### 3.1. Search Results

Figure S1 presents a flowchart outlining the screening and selection of the included studies. A total of 1085 references were obtained from the three databases, trial registry websites, and a manual examination of bibliographies. Among these, we excluded 261 duplicate studies and 766 studies with obviously irrelevant titles and abstracts. The remaining 58 studies underwent full-text screening, and five randomized controlled trials (RCTs) from 2019 to 2021 and three retrospective studies from 2015 to 2019 were included in the final meta-analysis.

### 3.2. Study Characteristics

The basic characteristics of the included studies are outlined in Table 1. A total of 1178 participants (0.01% atropine group, 600; control group, 578) were included. All RCTs [14,19,20,27,28] were conducted in Asian countries (Hong Kong, India, Japan, and China), while the retrospective studies [35,36,37] enrolled European or multi-ethnic participants and were performed in Italy [36] or the United States [35,37]. Among the included studies, one RCT [28] and one retrospective study [35] had follow-up data for 2 years, while the others provided 1-year follow-up data. In the case of multi-arm studies [14,19], we only extracted data from the 0.01% atropine and control groups. Of note, Fu et al. [19] did not report the results of pupil diameter as photopic or mesopic, and the lighting level in that study was kept in the range of 300 to 310 lux. Thus, we pooled the outcome data as the change in photopic pupil diameter.

### 3.3. Risk of Bias Assessment

Most domain-level judgments in the enrolled RCTs indicated a low risk of bias. The detailed risk of bias for the enrolled RCTs is reported in Table S3. The assessment revealed a moderate overall risk of bias in three non-RCTs (see details in Table S4).

### 3.4. Pooled Effects of the Efficacy Outcome

#### 3.4.1. Spherical Equivalent Refractive Error

**Figure 1:**
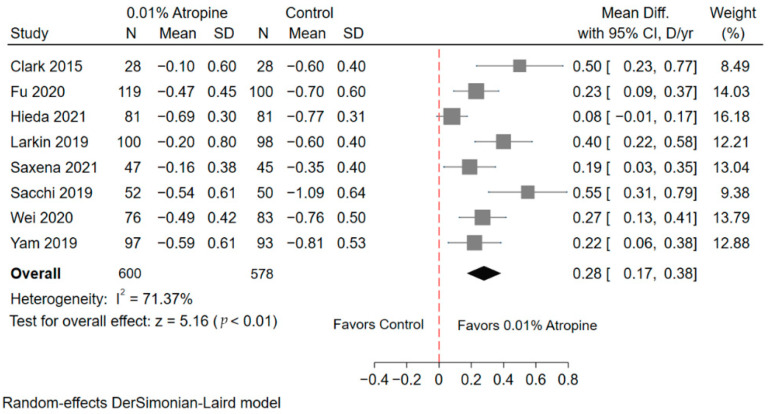
Forest plot of standardized equivalent refraction between the 0.01% atropine and control groups. SD, standard deviation; CI, confidence interval; D, diopter; yr, year.

Eight studies analyzed the change in SER at the 1-year follow-up (Figure 1). A total of 600 children received 0.01% atropine as treatment, and 578 children served as placebo group controls. The children who received 0.01% atropine showed significantly less progression in refraction than controls (weighted mean difference [WMD], 0.28 D per year; 95% confidence interval [CI] = 0.17 to 0.38; *p* < 0.01). Heterogeneity was significant (I^2^ = 71.37%). After removing the papers sequentially for sensitivity analysis, the WMD results were stable (Figure S2a). The pre-specified subgroups, according to study design, study population, mean baseline refraction, and mean baseline age demonstrated similar results, showing that 0.01% atropine significantly inhibited SER progression (Table 2). In subgroup of study population, the European (WMD, 0.55 D per year; 95% CI = 0.31, 0.79; *p* < 0.01) and multi-ethnicity groups (WMD, 0.43 D per year; 95% CI = 0.28, 0.58; *p* < 0.01) showed greater effect than the Asian groups (WMD, 0.18 D per year; 95% CI = 0.11, 0.26; *p* < 0.01). After stratifying age at 10 or mean baseline refraction at −3.00 D, patients at age >10 group or mean baseline refraction >−3.00 D group seemingly demonstrated greater effect.

#### 3.4.2. Axial Length

**Figure 2:**
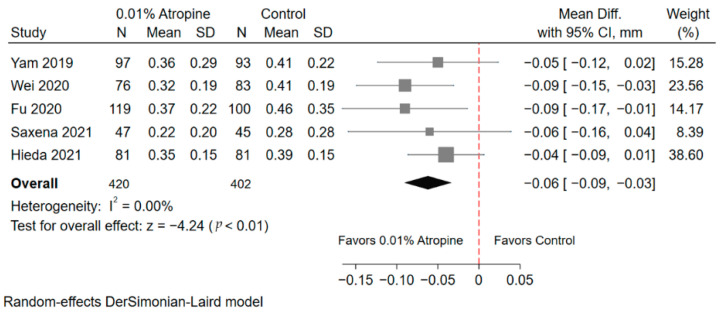
Forest plot of axial length between the 0.01% atropine and control groups. SD, standard deviation; CI, confidence interval.

Five RCTs reported the value of AL elongation at the 1-year follow-up (Figure 2). A total of 420 children received 0.01% atropine as treatment, and 402 children served as placebo group controls. The AL elongation of the 0.01% atropine group was significantly slower than that of the controls (WMD, −0.06 mm; 95% CI = −0.09, −0.03; *p* < 0.01). The overall heterogeneity I^2^ was 0%. After omitting the papers individually in sensitivity analysis, the WMDs were similar to the above findings (Figure S2b).

### 3.5. Pooled Effects of the Safety Outcome

#### 3.5.1. Accommodative Amplitude

**Figure 3:**
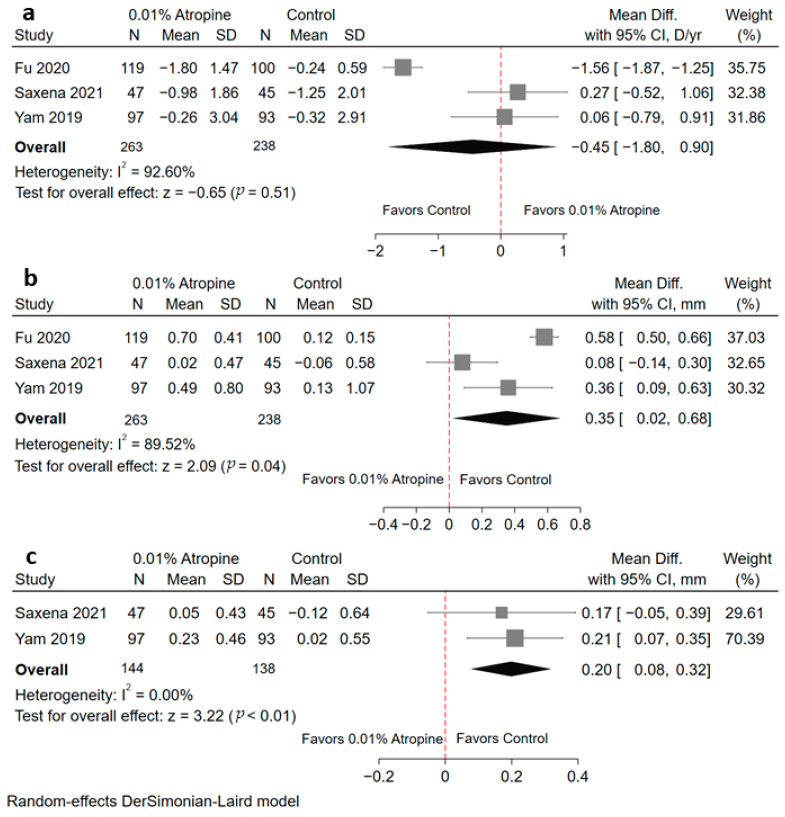
Forest plot of safety profiles between the 0.01% atropine and control groups; (**a**) change in accommodative amplitude, (**b**) change in photopic pupil diameter, (**c**) change in mesopic pupil diameter. Diff, difference; SD, standard deviation; CI, confidence interval; D, diopter; yr, year.

Three RCTs (including 501 patients) were included (Figure 3a). Children with myopia treated with 0.01% atropine did not show significantly lower accommodative amplitudes than the controls (WMD, −0.45 mm; 95% CI = −1.80, 0.90; *p* = 0.51). Significant heterogeneity was noted (I^2^ = 92.60%). Of note, after omitting Fu et al. [17], the heterogeneity was significantly reduced (I^2^= 0%), but the result still showed no statistical significance (WMD, 0.17 mm; 95% CI = −0.41, 0.75; *p* = 0.56) (Figure S2c).

#### 3.5.2. Photopic Pupil Diameter

Three RCTs (including 501 patients) were analyzed (Figure 3b). Children with myopia who received 0.01% atropine showed significantly increased in photopic pupil diameter (WMD, 0.35 mm; 95% CI = 0.02, 0.68; *p* = 0.04). High heterogeneity was detected (I^2^ = 89.52%; *p* < 0.01). After removing Saxena et al. [18], the heterogeneity decreased significantly (I^2^ = 58%), the photopic pupil diameter was still increased (WMD, 0.51 mm; 95% CI = 0.31, 0.71; *p* < 0.01) (Figure S2d).

#### 3.5.3. Mesopic Pupil Diameter

Only two RCTs provided complete data of mesopic pupil diameter (Figure 3c). A total of 282 children (144 in the 0.01% atropine group and 138 in the control group) were included. Significant increased mesopic pupil diameter was noted in the 0.01% atropine group (WMD, 0.20 mm; 95% CI = 0.08, 0.32; *p* < 0.01). No significant heterogeneity was detected (I^2^ = 0%).

### 3.6. Modified Hartung–Knapp–Sidik–Jonkman (HKSJ) Sensitivity Analysis

The overall effects on each outcome before and after modified HKSJ adjustment are presented in Table S5. Overall, the adjusted results in efficacy outcomes were similar to those from our previous meta-analyses, which indicates that our pooled effects were robust. However, the pooled results of the safety profiles showed a non-significant increase in photopic and mesopic pupil diameter after the modified HKSJ adjustment.

## 4. Discussion

Our meta-analysis collected up to date information and demonstrated that 0.01% atropine is effective in retarding childhood myopia progression, as measure by SER and AL over a period of 1 year. Regarding safety outcomes, there was no significant change in accommodative amplitude between 0.01% atropine and controls at the 1-year follow-up. Although both photopic and mesopic pupil diameter showed a significant increase in the 0.01% atropine group compared with controls, the clinical impacts of this phenomenon may be subtle (with an upper confidence interval of photopic and mesopic pupil diameter of 0.68 mm and 0.32 mm, respectively). In our subgroup analysis of SER, myopic children with lower baseline refraction (>−3 D) and older age (>10-year-old) obtained better responses with 0.01% atropine treatment. European and multi-ethnicity groups showed greater effect than Asian groups.

In 2016, a network meta-analysis [38] revealed that 0.01% atropine has a moderate efficacy in suppressing SER and AL progression (SER = 0.53 D/year, CI = 0.21 to 0.85; AL = −0.25 mm/year, CI = −0.25 to −0.05). However, no RCTs directly compared the 0.01% atropine and controls, and the findings were completely derived from indirect evidence. In 2017, Gong et al. [39] evaluated different doses of atropine (0.01% to 1%) to treat childhood myopia in a meta-analysis. Although they found 0.01% atropine was effective in slowing rates of SER progression (WMD, 0.50; CI = 0.24 to 0.76), only one retrospective study regarding 0.01% atropine was enrolled, and no information about AL changes was reported. Recently, one retrospective analysis of 13 myopic Australian children reported 0.01% atropine did not inhibit axial growth in ‘fast’ progressors compared to the age-matched untreated myope model (0.265 vs. 0.245 mm/year, *p* = 0.754, Power = 0.8) [40]. Our present meta-analysis used the latest evidence, including eight studies (five RCTs and three retrospective studies), and found a significant effect of 0.01% atropine in inhibiting myopic progression (SER = 0.28 D/year, CI = 0.17 to 0.38; AL = −0.06 mm/year, CI = −0.09 to −0.03). Our subgroup analysis identified a larger effect of 0.01% atropine in users with a mean age >10 years compared with users <10 years. This finding was consistent with the Low-Concentration Atropine for Myopia Progression (LAMP) Study [22]. The elongation of AL slowed and stabilized in older children might be part of the reason. Furthermore, patients with lower base line refraction (>−3 D) obtained better responses than those with higher ones (<−3 D). Although the mechanism of this phenomenon was unclear, this information provides a useful guide for clinicians to find the ideal candidate for the use of 0.01% atropine in myopic control.

The issue regarding the optimal dose of atropine has recently been up for debate. Two studies [38,39] recommended 0.01% atropine for myopic control due to its high acceptability. Of note, the long-term efficacy and safety profiles of 0.01% atropine have been proved in well-established ATOM2 trials [17]; a double-blind design and a large cohort of subjects (400 in each study) demonstrated that 0.01% atropine for periods up to 5 years is a clinically viable treatment of myopia with the best-sustained effect on myopia retardation. Compared to placebo, 0.01% atropine also demonstrated significant effect over a 2-year period [28,35]. However, several studies investigating the efficacy of 0.01% atropine for myopia control have produced inconsistent findings in AL change [14,19,20,27,28]. For example, Saxena et al. [20] and Yam et al. [14] found a non-significant efficacy of 0.01% atropine for retarding axial elongation at 1-year follow-up. In contrast, the efficacy of 0.01% in AL inhibition was identified in an RCT involving a large sample size (280 children) [19]. The present meta-analysis pooled axial elongation results from five high-quality RCTs, including 420 participants in the 0.01% atropine group and 402 in the control group, showing a significant efficacy of 0.01% atropine for childhood myopia. In addition, ATOM2 trial [17] demonstrated the significantly lower rebound of axial length for 0.01% atropine (0.19 ± 0.13 mm) compared to 0.5% and 0.1% atropine (0.35 ± 0.20 mm and 0.33 ± 0.18 mm, respectively, *p* < 0.001). This finding may instill confidence in practitioners and patients using 0.01% atropine.

An evaluation of the benefit versus risks will help better characterize the value of atropine in clinical practice to slow myopia. In the present meta-analysis, we evaluated the safety profiles of 0.01% atropine eye drops by quantifying the changes in accommodative amplitude, photopic, and mesopic pupil diameter. Although an increase in photopic and mesopic pupil diameter was noted in the 0.01% atropine group, the overall estimates were within the tolerable range [41,42]. The pooled estimates of change in accommodative amplitude were statistically insignificant and highly heterogeneous. This phenomenon may arise from different baseline accommodative amplitude and age as well as different measuring methods. Of note, we reviewed other common adverse events such as poor near visual acuity and allergic conjunctivitis in our included studies, and no significant influences were noted. Moreover, the drop-out rates in the enrolled studies were generally below 20%, and no treatment-related severe adverse events were noted, which indicates the high applicability of 0.01% atropine in clinical practice.

Phase 2 of the LAMP study [43] reported that the 0.05% atropine has a better effect in myopic control compared with 0.025% and 0.01% atropine. However, 31.2% of 0.05% atropine user developed photophobia at two weeks, which is significantly greater than 0.01% atropine users (5.5%), and its long-term safety profile (>2 years) and rebound phenomenon were unclear. By using a <3 mm increase in photopic pupil size as the cutoff beyond which there will be significant discomfort for some users [41], the reported data from Sankaridurg et al. [42] showed that some eyes would reach this cutoff in 0.025% and 0.05% atropine users; with 0.01% atropine, the change in photopic pupil size was approximately 1 mm and appears in alignment with the efficacy data. In a 3 × 3 phase I clinical trial paradigm, Cooper et al. [41] also concluded that 0.02% atropine might be the highest concentration that does not produce significant clinical symptoms from accommodation paresis or pupillary dilation. In addition, some real-world data [35,36,40] revealed that 0.01% atropine slows the rate of myopia progression in non-Asian patients with favorable safety profiles. Joachimsen et al. [44] even reported that 0.05% atropine induced significantly more anisocoria (2.9 mm compared to 0.8 mm) and loss of accommodation amplitude (loss of 4.2 D compared to 0.05 D) in Caucasian children compared to 0.01% atropine. They supposed that high variation in iris color and the affinity of atropine for melanin might be speculated for the differences [45], and this phenomenon was observed by Myles et al. [40]; those with blue eyes were more susceptible to experiencing dilated pupils as a consequence of atropine treatment. Loughman et al. [46] also proved 0.01% atropine to be a viable therapeutic option among Caucasian eyes. In our subgroup analysis of the study population, the results also demonstrated that 0.01% atropine was a somewhat more effective treatment in non-Asians than in Asians for SER progression. This finding is particularly meaningful since a previous meta-analysis [47] revealed that atropine slows myopia progression more in Asian than non-Asian children. The current evidence for slowing myopia with concentrations of atropine greater than 0.01% is promising, but it is not sufficiently clear that the profile is favorable when it comes to side effects [41]. Taken together, we asserted that 0.01% atropine is useful for myopic control due to its evidence-based long-term effect and applicability in the general population. Further clinical trials are still needed to explore the applicability of this treatment in non-Asian populations.

The major strength of the present study was the inclusion of high-quality RCTs that provided valuable primary data. Further, the overall heterogeneity of the pooling data in AL was low, and the significant results were robust after the leave-one-out and the modified HKSJ adjustment sensitivity analyses. This finding can resolve the inconsistency found in previous studies. Furthermore, we systematically summarized evidence regarding 0.01% atropine regardless of Asian or non-Asian population, providing helpful information for clinicians.

There are several limitations to this study. First, most of our included studies had short-term follow-up periods (1 year in six studies and 2 years in two studies). The long-term efficacy and safety profiles of 0.01% atropine eye drops cannot be obtained from this study. Second, we cannot directly compare the benefit–risk ratio between 0.01% atropine and other low dose atropine (such as 0.05% and 0.025%) in this study. However, currently, there was only one trial that compared those doses of atropine directly [14]. We look forward to collecting more relevant evidence and providing helpful information. Lastly, we did not conduct a meta-regression to assess the association between baseline characteristics and myopia progression after 0.01% atropine treatment since the power may be insufficient to identify the potential effect.

## 5. Conclusions

In conclusion, our meta-analysis demonstrated that 0.01% atropine had a favorable efficacy and adequate safety for managing childhood myopia over a 1-year period. The children who received 0.01% atropine showed significantly less progression in axial length and refraction than controls. 0.01% atropine also has a better treatment effect in children with lower refractive error and older age and seems more effective in non-Asian patients. Myopic children who have photophobia and blurry near vision after administration of higher-dose atropine may benefit with 0.01% atropine treatment. Further studies are warranted to elucidate the long-term efficacy and safety of 0.01% atropine eye drops and their applicability in different ethnic groups.

## Figures and Tables

**Table 1:** Characteristics of studies included in the meta-analysis.

Study (Author, Year)	Country	Study Design	Study Population	Follow- Up, yr	Intervention	Number of Eyes	Mean Age (SD), yr	Mean Baseline Refraction (SD), D	Mean Baseline Axial Length (SD), mm	Drop-Out Rate
Clark 2015	United states	Retrospective Case-control	Multi-ethnicity	1.1 (0.3)	Placebo 0.01% atropine	28 28	10.2 (2.2) 10.2 (2.2)	−2.0 (1.5) −2.0 (1.6)	NA NA	NA NA
Fu 2020	China	RCT	Asian	1	Placebo 0.01% atropine	100 119	9.5 (1.4) 9.3 (1.9)	−2.68 (1.42) −2.70 (1.64)	24.55 (0.71) 24.58 (0.74)	20/120 23/142
Heida 2021	Japan	RCT	Asian	2	Placebo 0.01% atropine	80 * 78 *	8.98 (1.50) 8.99 (1.44)	R/L: −2.96 (1.24)/ −2.97 (1.22) R//L: −2.92 (1.43)/ −2.90 (1.38)	R:/L: 24.50 (0.69)/ 24.48 (0.70) R:/L: 24.41 (0.86)/ 24.40 (0.87)	6/86 * 7/85 *
Larkin 2019	United states	Retrospective Case-control	Multi-ethnicity	2	Placebo 0.01% atropine	98 100	9.2 (2.11) 9.3 (2.10)	−2.8 (1.6) −3.1 (1.9)	NA NA	NA NA
Saxena 2021	India	RCT	Asian	1	Placebo 0.01% atropine	45 47	10.8 (2.2) 10.6 (2.2)	−3.71 (1.37) −3.38 (1.32)	24.70 (0.80) 24.60 (1.02)	5/50 3/50
Sacchi 2019	Italy	Retrospective Cohort	European	1	Placebo 0.01% atropine	50 52	12.1 (2.9) 9.7 (2.3)	−2.63 (2.68) −3.00 (2.23)	NA NA	NA NA
Wei 2020	China	RCT	Asian	1	Placebo 0.01% atropine	83 76	9.84 (1.53) 9.44 (1.80)	−2.64 (1.46) −2.52 (1.33)	24.69 (0.97) 24.50 (0.76)	27/110 34/110
Yam 2019	Hong Kong	RCT	Asian	1	Placebo 0.01% atropine	93 97	8.42 (1.72) 8.23 (1.83)	−3.85 (1.95) −3.77 (1.85)	24.82 (0.97) 24.70 (0.99)	18/111 13/110

Abbreviations: yr, year; SD: standardized deviation; D, diopter; RCT: randomized controlled trial; NA: not applicable; R: right eye; L: left eye. * Two-year follow-up data presented.

**Table 2:** Subgroup analyses of efficacy outcomes in standardized equivalent refraction.

	Standardized Equivalent Refraction (SER)			
Subgroups	No. of Studies	Pooled MD (95% CI)	*p*-Value	I^2^ (%)
Overall	8	0.28 (0.17 to 0.38)	<0.01 **	71.4
Study design				
RCTs	5	0.18 (0.11 to 0.26)	<0.01 **	38.5
Non-RCTs	3	0.46 (0.34 to 0.59)	<0.01 **	0.0
Study population				
Asian only	5	0.18 (0.11 to 0.26)	<0.01 **	38.5
European only	1	0.55 (0.31 to 0.79)	<0.01 **	-
Multi-ethnicity	2	0.43 (0.28 to 0.58)	<0.01 **	0.0
Mean age, year				
Age < 10	5	0.23 (0.12 to 0.34)	<0.01 **	67.5
Age > 10	3	0.40 (0.15 to 0.65)	<0.01 **	73.9
Mean baseline refraction, Diopter				
>−3.00	6	0.31 (0.17 to 0.46)	<0.01 **	79.4
<−3.00	2	0.20 (0.09 to 0.32)	<0.01 **	0.0

Abbreviations: MD, mean difference; CI, confidence interval; RCT: randomized controlled trial. ** *p* < 0.01.

## Data Availability

Data supporting the findings of this study are available within the included articles or published studies.

## Supplementary Materials

The following are available online at https://www.mdpi.com/article/10.3390/jcm10173766/s1, Figure S1: Preferred reporting items for systematic reviews and meta-analyses (PRISMA) flow diagram of the literature search process, Table S1: PRISMA checklist, Figure S2: Leave-one-out sensitivity analysis of efficacy and safety outcome, Table S2: Search strategy modified in PubMed (a), Embase (b), Cochrane CENTRAL (c), Table S3: Summary of ROB 2.0 assessment in RCTs, Table S4: Summary of ROBINS-I assessment in non—RCTs, Table S5: Sensitivity analysis of overall effects of each outcome before and after modified Hartung–Knapp–Sidik–Jonkman (HKSJ) adjustment.
