# Supplementary material for: Is 0.01% Atropine an Effective and Safe Treatment for Myopic Children? A Systemic Review and Meta-Analysis
Source: J Clin Med. 2021 Aug 24;10(17):3766. doi: 10.3390/jcm10173766 (PMC8432260; doi:10.3390/jcm10173766)
Supplement: Supplementary file 1 [file jcm-10-03766-s001.zip › jcm-1318609-supplementary.pdf]

Supplementary Online Contents

Figure S1. Preferred Reporting Items for Systematic Reviews and Meta-Analyses (PRISMA) flow diagram of the literature search process

Table S1. PRISMA checklist

Figure S2. Leave-one-out sensitivity analysis of efficacy and safety outcome

Table S2. Search strategy modified in PubMed (a), Embase (b), Cochrane CENTRAL (c)

Table S3. Summary of ROB 2.0 assessment in RCTs

Table S4. Summary of ROBINS-I assessment in non – RCTs

Table S5. Sensitivity analysis of overall effects of each outcome before and after modified Hartung-Knapp-Sidik-Jonkman (HKSJ) adjustment

Figure S1. Preferred Reporting Items for Systematic Reviews and Meta-Analyses (PRISMA) flow diagram of the literature search process

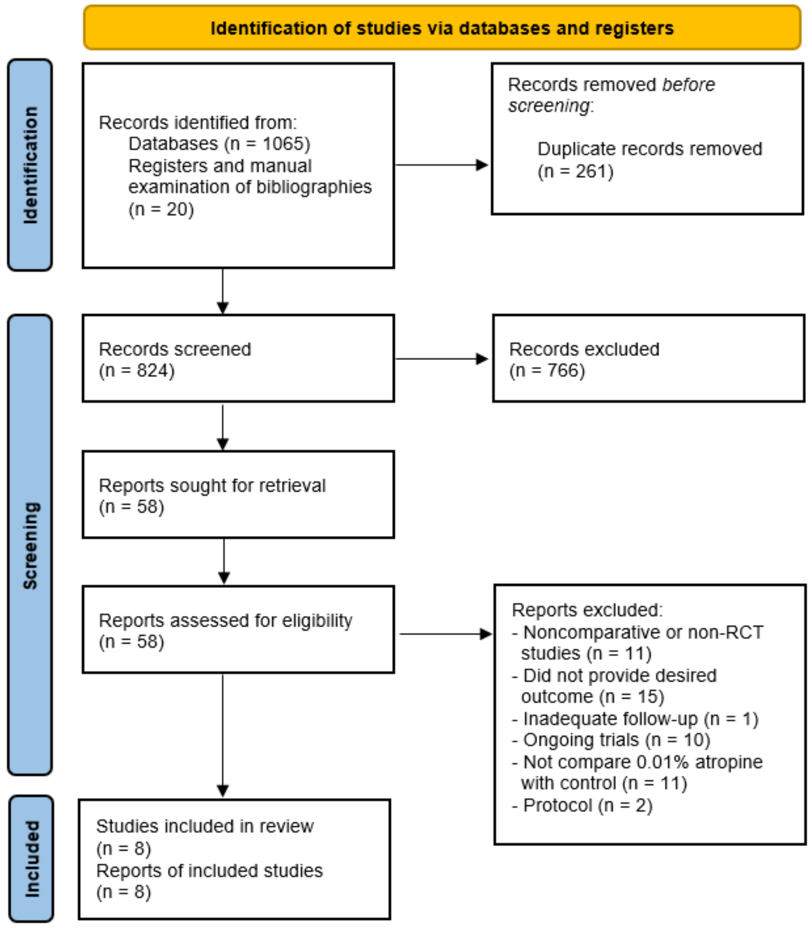

Table S1. PRISMA checklist

Section and Topic	Item #	Checklist item	Location where item is reported
**TITLE**			
Title	1	Identify the report as a systematic review.	P1 (Page 1.)
**ABSTRACT**			
Abstract	2	See the PRISMA 2020 for Abstracts checklist.	P1
**INTRODUCTION**			
Rationale	3	Describe the rationale for the review in the context of existing knowledge.	P1-2
Objectives	4	Provide an explicit statement of the objective(s) or question(s) the review addresses.	P2
**METHODS**			
Eligibility criteria	5	Specify the inclusion and exclusion criteria for the review and how studies were grouped for the syntheses.	P3
Information sources	6	Specify all databases, registers, websites, organisations, reference lists and other sources searched or consulted to identify studies. Specify the date when each source was last searched or consulted.	P2-3
Search strategy	7	Present the full search strategies for all databases, registers and websites, including any filters and limits used.	Supplement Table S2.
Selection process	8	Specify the methods used to decide whether a study met the inclusion criteria of the review, including how many reviewers screened each record and each report retrieved, whether they worked independently, and if applicable, details of automation tools used in the process.	P3

Section and Topic	Item #	Checklist item	Location where item is reported
Data collection process	9	Specify the methods used to collect data from reports, including how many reviewers collected data from each report, whether they worked independently, any processes for obtaining or confirming data from study investigators, and if applicable, details of automation tools used in the process.	P3
Data items	10a	List and define all outcomes for which data were sought. Specify whether all results that were compatible with each outcome domain in each study were sought (e.g. for all measures, time points, analyses), and if not, the methods used to decide which results to collect.	P3
	10b	List and define all other variables for which data were sought (e.g. participant and intervention characteristics, funding sources). Describe any assumptions made about any missing or unclear information.	P3
Study risk of bias assessment	11	Specify the methods used to assess risk of bias in the included studies, including details of the tool(s) used, how many reviewers assessed each study and whether they worked independently, and if applicable, details of automation tools used in the process.	P3
Effect measures	12	Specify for each outcome the effect measure(s) (e.g. risk ratio, mean difference) used in the synthesis or presentation of results.	P3
Synthesis methods	13a	Describe the processes used to decide which studies were eligible for each synthesis (e.g. tabulating the study intervention characteristics and comparing against the planned groups for each synthesis (item #5)).	P3 Table1
	13b	Describe any methods required to prepare the data for presentation or synthesis, such as handling of missing summary statistics, or data conversions.	P3
	13c	Describe any methods used to tabulate or visually display results of individual studies and syntheses.	Nil
	13d	Describe any methods used to synthesize results and provide a rationale for the choice(s). If meta-analysis was performed, describe the model(s), method(s) to identify the presence and extent of statistical heterogeneity, and software package(s) used.	P3
	13e	Describe any methods used to explore possible causes of heterogeneity among study results (e.g. subgroup analysis, meta-regression).	P3
	13f	Describe any sensitivity analyses conducted to assess robustness of the synthesized results.	P3
Reporting bias	14	Describe any methods used to assess risk of bias due to missing results in a synthesis (arising from reporting biases).	P3

Section and Topic	Item #	Checklist item	Location where item is reported
assessment			
Certainty assessment	15	Describe any methods used to assess certainty (or confidence) in the body of evidence for an outcome.	Nil
**RESULTS**			
Study selection	16a	Describe the results of the search and selection process, from the number of records identified in the search to the number of studies included in the review, ideally using a flow diagram.	P3-4<br>Supplement Figure S1
	16b	Cite studies that might appear to meet the inclusion criteria, but which were excluded, and explain why they were excluded.	Supplement Figure S1
Study characteristics	17	Cite each included study and present its characteristics.	P4<br>Table 1.
Risk of bias in studies	18	Present assessments of risk of bias for each included study.	Supplement Table S3-4
Results of individual studies	19	For all outcomes, present, for each study: (a) summary statistics for each group (where appropriate) and (b) an effect estimate and its precision (e.g. confidence/credible interval), ideally using structured tables or plots.	P7-8
Results of syntheses	20a	For each synthesis, briefly summarise the characteristics and risk of bias among contributing studies.	P6-8
	20b	Present results of all statistical syntheses conducted. If meta-analysis was done, present for each the summary estimate and its precision (e.g. confidence/credible interval) and measures of statistical heterogeneity. If comparing groups, describe the direction of the effect.	P6-8<br>Fig.1-3
	20c	Present results of all investigations of possible causes of heterogeneity among study results.	P6-8
	20d	Present results of all sensitivity analyses conducted to assess the robustness of the synthesized results.	P6-9,<br>Figure S2.<br>Table S5.
Reporting biases	21	Present assessments of risk of bias due to missing results (arising from reporting biases) for each synthesis assessed.	Nil

Section and Topic	Item #	Checklist item	Location where item is reported
Certainty of evidence	22	Present assessments of certainty (or confidence) in the body of evidence for each outcome assessed.	Nil
**DISCUSSION**			
Discussion	23a	Provide a general interpretation of the results in the context of other evidence.	P9
	23b	Discuss any limitations of the evidence included in the review.	P10-11
	23c	Discuss any limitations of the review processes used.	P10-11
	23d	Discuss implications of the results for practice, policy, and future research.	P11
**OTHER INFORMATION**			
Registration and protocol	24a	Provide registration information for the review, including register name and registration number, or state that the review was not registered.	P2
	24b	Indicate where the review protocol can be accessed, or state that a protocol was not prepared.	P2
	24c	Describe and explain any amendments to information provided at registration or in the protocol.	Nil
Support	25	Describe sources of financial or non-financial support for the review, and the role of the funders or sponsors in the review.	Nil
Competing interests	26	Declare any competing interests of review authors.	Nil
Availability of data, code and other materials	27	Report which of the following are publicly available and where they can be found: template data collection forms; data extracted from included studies; data used for all analyses; analytic code; any other materials used in the review.	Nil

Figure S2. Leave-one-out sensitivity analysis of efficacy and safety outcome

Figure S2a. Leave-one-out sensitivity analysis of standardized equivalent refractive errors

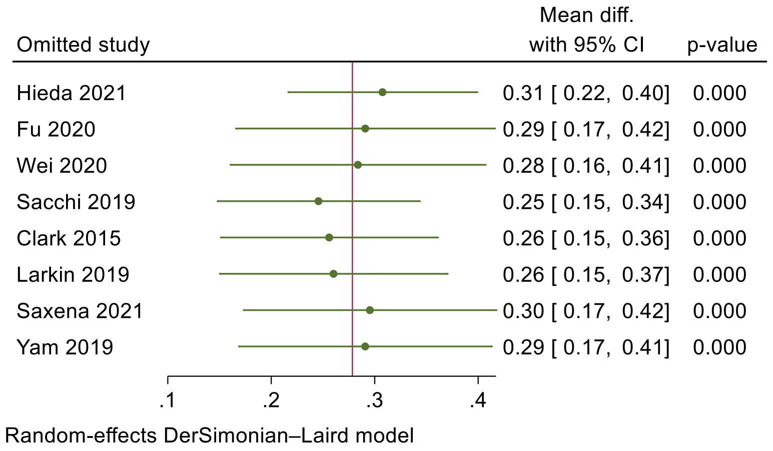

Figure S2b. Leave-one-out sensitivity analysis of axial length

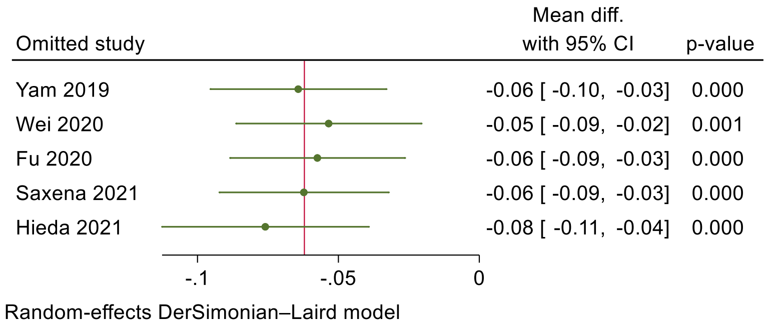

Figure S2c. Leave-one-out sensitivity analysis of accommodative amplitude

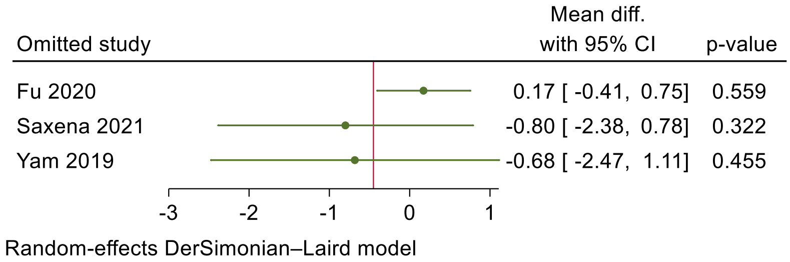

Figure S2d. Leave-one-out sensitivity analysis of photopic pupil diameter

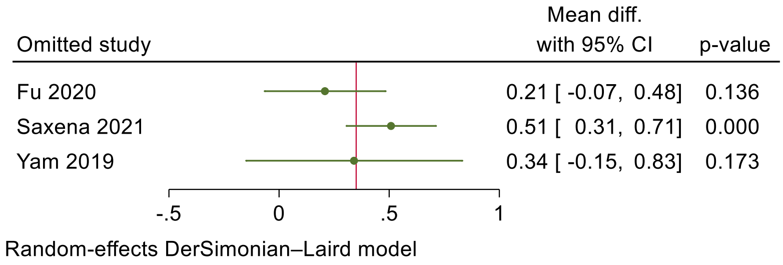

Table S2. Search strategies modified in PubMed (a), Embase (b), and Cochrane CENTRAL (c)

a. Search strategy in PubMed; search date: 2021/06/27)

#	Search syntax	Citations found
1	((("short"[All Fields] OR "shorts"[All Fields] OR "near*"[All Fields]) AND "sight*"[All Fields]) OR "myop*"[All Fields] OR "myopia"[MeSH Terms])	
2	("refractive errors"[MeSH Terms] OR "refract*"[All Fields] OR "accommodation, ocular"[MeSH Terms] OR "visual acuity"[MeSH Terms] OR (("axial"[All Fields] OR "axially"[All Fields] OR "axials"[All Fields]) AND ("length"[All Fields] OR "lengths"[All Fields])) OR ("progress*"[All Fields] OR "slow*"[All Fields] OR "retard*"[All Fields] OR "funct*"[All Fields] OR "prevent*"[All Fields]))	
3	"atropine*"[All Fields]	
4	1 AND 2 AND 3	389

b. Search strategy in Embase; search date: 2021/06/27)

#	Search syntax	Citations found
1	exp myopia/	
2	myop$.tw.	
3	((short or near$) adj3 sight$).tw.	
4	or/1-3	
5	exp refraction error/	
6	refract$.tw.	
7	exp axial length/	
8	exp accommodation/	
9	or/5-8	
10	exp atropine/	
11	4 and 9 and 10	
12		619

c. **Search strategy in Cochrane Central Register of Controlled Trials (CENTRAL; search date: 2021/06/27)**

#	Search syntax	Citations found
1	MeSH descriptor myopia	
2	(short OR near*) next sight*	
3	myop*	
4	#1 OR #2 OR #3	
5	MeSH descriptor Atropine	
6	MeSH descriptor Refractive Errors	
7	refract*	
8	MeSH descriptor Axial length, Eye	
9	progress* or slow* or retard* or funct*	
10	MeSH descriptor Accommodation, Ocular	
11	#6 OR #7 OR #8 OR #9 OR #10	
12	#4 AND #5 AND #11	
13		57

Table S3. Summary of ROB 2.0 assessment in RCTs

Study							
**First author**	**Year**	**Bias arising from randomization process**	**Bias due to deviations from intended intervention**	**Bias due to missing outcome data**	**Bias in measurement of the outcome**	**Bias in selection of the reported result**	**Overall bias**
**Fu**	**2020**	Low	Some concern	Some concern	Low	Low	Some concern
**Hieda**	**2021**	Low	Low	Low	Low	Low	Low
**Saxena**	**2021**	Low	Low	Low	Low	Low	Low
**Wei**	**2020**	Low	Low	Some concern	Some concern	Low	Some concern
**Yam**	**2019**	Some concern	Low	Low	Low	Low	Some concern

Table S4. Summary of ROBINS-I assessment in non - RCTs

Study		Pre-intervention		At intervention	Post-intervention				Overall risk of bias
First author	Year	Bias due to confounding	Bias in selection of participants into the study	Bias in classification of interventions	Bias due to deviations from intended interventions	Bias due to missing data	Bias in measurement of outcomes	Bias in selection of the reported result	low / moderate / serious / critical
**Clark**	**2015**	moderate	moderate	Low	Low	Low	moderate	Low	moderate
**Larkin**	**2019**	moderate	Low	Low	moderate	Low	moderate	Low	moderate
**Sacchi**	**2019**	moderate	Low	Low	moderate	Low	Low	Low	moderate

Table S5. Sensitivity analysis of overall effects of each outcome before and after modified Hartung-Knapp-Sidik-Jonkman (HKSJ) adjustment

Outcome	No. of studies	Pooled WMD (95%CI)	*P*-value	I^2 (%)
**SER**				
Before adjustment	8	0.28 (0.17 to 0.38)	< 0.01**	39
After adjustment	8	0.28 (0.15 to 0.41)	< 0.01**	39
**AL**				
Before adjustment	5	- 0.06 (- 0.03 to – 0.09)	< 0.01**	0
After adjustment	5	- 0.06 (- 0.03 to – 0.09)	0.01*	0
**Photopic pupil size**				
Before adjustment	3	0.35 (0.02 to 0.68)	0.04*	90
After adjustment	3	0.35 (- 0.28 to 0.98)	0.14	90
**Mesopic pupil size**				
Before adjustment	2	0.20 (0.08 to 0.32)	<0.01**	0
After adjustment	2	0.20 (- 0.03 to 0.43)	0.06	0

SER: Standardized equivalent refractive errors; AL: Axial length

*p < 0.05; **p < 0.01
